# Association of rhinitis with asthma prevalence and severity

**DOI:** 10.1038/s41598-022-10448-w

**Published:** 2022-04-16

**Authors:** Antonio Acevedo-Prado, Teresa Seoane-Pillado, Angel López-Silvarrey-Varela, Francisco-Javier Salgado, María-Jesus Cruz, Ana Faraldo-Garcia, Juan-Jose Nieto-Fontarigo, Sonia Pértega-Díaz, J. Sanchez-Lastres, Miguel-Angel San-José-González, Luis Bamonde-Rodríguez, Luciano Garnelo-Suárez, Teresa Pérez-Castro, Manuel Sampedro-Campos, Francisco-Javier Gonzalez-Barcala

**Affiliations:** 1 Dirección Xeral de Atención Integral Sociosanitaria, Consellería de Política Social-Xunta de Galicia, Santiago de Compostela, Spain; 2 Área de Medicina Preventiva y Salud Pública, Departamento de Ciencias de La Salud, Universidade de A Coruña – A Coruña, A Coruña, Spain; 3 Fundación María José Jove, A Coruña, Spain; 4 Departmento de Bioquímica Y Biología Molecular, Facultad de Biología-Centro de Investigacións Biolóxicas (CIBUS), Universidade de Santiago de Compostela, Santiago de Compostela, Spain; 5 Traslational Research In Airway Diseases Group (TRIAD), Instituto de Investigación Sanitaria de Santiago de Compostela (IDIS), Santiago de Compostela, Spain; 6 Servei de Pneumologia, Hospital Vall d Hebron, Barcelona, Spain; 7 CIBER Enfermedades Respiratorias-CIBERES, Instituto de Salud Carlos III, Madrid, Spain; 8 Departmento de Otorrinolaringología, Hospital Clínico-Universitario de Santiago de Compostela, Santiago de Compostela, Spain; 9 Respiratory Immunopharmacology Group, Department of Experimental Medical Science, Lund University, Lund, Sweden; 10 Unidad de Epidemiología Clínica y Bioestadística, Instituto de Investigación Biomédica de A Coruña, Complexo Hospitalario Universitario de A Coruña, A Coruña, Spain; 11 Pediatría-Servicio Galego de Saúde, A Coruña, Spain; 12 Grupo de Investigación Cardiovascular (GRINCAR), Instituto de Investigación Biomédica de A Coruña (INIBIC), Instituto Universitario de Ciencias de La Salud, Universidade da Coruña, A Coruña, Spain; 13 Servicio de Neumología-Hospital Clínico Universitario de Santiago de Compostela, A Coruña, Spain; 14 Departamento de Medicina, Universidad de Santiago de Compostela, A Coruña, Spain

**Keywords:** Asthma, Risk factors, Epidemiology, Comorbidities, Respiratory signs and symptoms

## Abstract

Asthma and rhinitis often co-exist in the same patient. Although some authors observed a higher prevalence and/or greater severity of asthma in patients with rhinitis, this view is not homogeneous and the debate continues. The aim of our study is to describe the prevalence of rhinitis in children and adolescents and to analyse their relationship with the prevalence of asthma. A multicentre study was conducted using the methodology of the International Study of Asthma and Allergies in Childhood (ISAAC). The target population of the study was all those school children aged 6–7 and 13–14 years from 6 of the main health catchment areas of Galicia (1.9 million inhabitants). The schools required were randomly selected, and all children in the targeted age ranges were included. Multiple logistic regression was used to obtain adjusted prevalence odds ratios (OR) between asthma symptoms of the schoolchildren and rhinitis prevalence. The results were adjusted for parental smoking habits, maternal education level, cat and dog exposure, and obesity. A total of 21,420 valid questionnaires were finally obtained. Rhinitis was associated with a significant increase in the prevalence of asthma in both age groups. The highest OR were 11.375 for exercise induced asthma (EIA) for children with recent rhinoconjunctivitis and 9.807 for children with recent rhinitis in 6–7 years old group. The prevalence OR’s are higher in EIA and severe asthmatics. Rhinitis in children and adolescents is associated with a higher prevalence and severity of asthma.

## Introduction

Asthma is a chronic inflammatory disease that often affects children and adolescents. Its prevalence varies widely between different geographic zones, being around 2%–4% in Asia, Northern Africa, Eastern Europe, and Eastern Mediterranean regions, whilst in other countries in Southeast Asia, North America, and Latin America it reaches 29%–32%^1^. Our community, Galicia, in the Northwest of Spain, has a medium prevalence compared to the rest of the world, being between 11.4% and 15.7% in children and between 8.8% and 18.8% in adolescents^2^. It is one of the most common causes of Disability-adjusted life years in children and young adults^1^. The mortality due to asthma in this age group has gradually reduced in the last few years, although with significant differences between different countries^1,3^.

Rhinitis is diagnosed based on the presence of typical symptoms, such as rhinorrhoea, nasal obstruction, sneezing, or itching. In order to define chronic rhinitis, two of these symptoms must be present for at least one hour per day for 12 weeks a year. It is a very common illness in the paediatric population, with a prevalence that reaches 45% in some areas^4^.

Asthma prevalence varies significantly worldwide and even between regions closely related geographically and ethnically^2,5,6^.

Asthma and rhinitis often co-exist in the same patient, in children as well as in adults. Asthma is found in up to 38% of patients with allergic rhinitis (AR) and nasal symptoms are present in 6% to 85% patients with asthma^7^. However, although some authors observed a higher prevalence and/or greater severity of asthma in patients with rhinitis^8,9^, this was not confirmed by other studies^10,11^. Likewise, although different studies appear to support the atopic course, as such that allergic diseases occur following a time-based order from atopic dermatitis and food allergy to asthma and allergic rhinitis^12,13^, other authors say that the typical atopic course does not occur in their populations, possibly in relation to environmental factors^14,15^. The results of previous studies seem to support that the atopic march is less frequent than classically considered. In a UK cohort study, including more than 9000 children, it was observed that less than 7% of children with eczema follow the trajectory of the classic atopic march^16^. In another study, it has been shown that a fifth of children with asthma without food allergy or atopic dermatitis ended up developing the dermatological disease, which we could be considered as a reverse atopic march^17^.

The aim of the present study is to describe the prevalence of rhinitis in children and adolescents in this community and to analyse the relationship with the prevalence and severity of asthma.

## Materials and methods

### Study population

A multicentre study was conducted using the methodology of the International Study of Asthma and Allergies in Childhood (ISAAC)^18^. Written questionnaires, previously translated and validated in Spanish, were used^19^. The study was approved by the Clinical Research Ethics Committee of Galicia (code number 2005/116). All research was performed in accordance with relevant guidelines/regulations, and in accordance with the Declaration of Helsinki. Informed consent was obtained from all subjects before inclusion.

The target population of the study was all those school children aged 6–7 and 13–14 years from 6 of the main health catchment areas of Galicia, which includes a total of 1.9 million inhabitants, sixty nine percent of the population in this Autonomous Region^19^. At these ages, schooling is mandatory by law, thus it can be assumed that the schooling rate is quite close to 100%. In each area, a research team leaded by a Medical Doctor specialist in Paediatrics or Respiratory Medicine was set up to carry-out the study.

Following the ISAAC protocol, a minimum sample size of 1000 valid questionnaires was established for each age group and for each area studied in order to obtain the levels of prevalence, and to also detect the possible differences between the areas analysed^18^.

The schools required from each health area were randomly selected, and all children in the targeted age ranges were included. The schools that refused to take part in the study were replaced with others. Permission was sought from parents or guardians, who also completed the questionnaires for the 6–7 years age group, whereas in the older age group the responses to the questionnaires were made by the children themselves.

Fieldwork was conducted between October 2006 and February 2007. The questionnaire data was introduced manually into a data base, using double entry with subsequent validation, in accordance with the ISAAC protocols. The environmental questionnaire included questions about asthma and rhinitis symptoms, self-reported height and weight, exposure to pets, smoking habits of parents, and mother’s education level. Obesity and overweight were defined in accordance with the Body Mass Index (BMI) cut-off points suggested by Cole et al.^20^ for each age group and gender. The educational level of the mother was classified into 3 categories: (1) No education or only primary school education; (2) Secondary school education; (3) University education. Four mutually exclusive categories of passive smoking were established for each child: neither parent smoked, the father only, the mother only, and both. The presence of a dog or cat in the home was classified based on the questionnaire questions corresponding to having a cat or dog in the home during the first year of life or during the past year.

For the purpose of this study several definitions of rhinitis were taken into account as independent variables. “Rhinitis ever” was defined as a positive answer to the question “Have you (has your child) ever had a problem with sneezing or a runny or blocked nose, when you (he or she) DID NOT have a cold or the flu?”. "Recent rhinitis" was determined by a positive response to question “In the past 12 months, have you (has your child) had a problem with sneezing or a runny or blocked nose, when you (he or she) DID NOT have a cold or the flu?”. "Recent rhinoconjunctivitis" determined by a combination of positive responses to questions: “In the past 12 months, have you (has your child) had a problem with sneezing or a runny or blocked nose, when you (he or she) DID NOT have a cold or the flu?”, and “In the past 12 months, has this nose problem been accompanied by itchy-watery eyes?”.

"Severe rhinoconjunctivitis" included children and adolescents with “Recent rhinoconjunctivitis” and, with the answer “a lot” to question “In the past 12 months, how much did this nose problem interfere with your (child´s) daily activities? (Not at all/a little/a moderate amount/a lot)”^19^.

As primary dependent variables, some definitions of asthma were considered. “Wheezing ever” was defined as a positive answer to the question “Has your child ever had wheezing or whistling in the chest at any time in the past?”. “Current asthma” was defined as a positive answer to the question “Has your child had wheezing or whistling in the chest during the last 12 months?”. “Severe asthma” was defined as a combination of the three questions assessing the severity of asthma: “How many attacks of wheezing has your child had during the last 12 months? (none, 1–3, 4–12, > 12)”, “In the last 12 months, how often on average has your child’s sleep been disturbed due to wheezing? (never, < 1 night/week, >  = 1 nights/week)”, and “In the last 12 months, has wheezing been severe enough to limit your child’s speech to only one or two words at a time between breaths?”. Children were considered to have current severe asthma when there were >  = 4 asthma attacks or when sleep was disturbed >  = 1 nights/week or when there had been an episode of speech limitation. “Exercise induced asthma (EIA)” was defined as a positive answer to the question, “In the last 12 months, has your child's chest sounded wheezy during or after exercise?”^20^.

### Data analysis

Children with rhinitis were considered as exposed, while those with asthma were considered as having the outcome of interest. Binary variables were compared using the Chi-squared test.

Adjusted Prevalence ORs and 95%CI were estimated using multivariate logistic regression models. For all statistical models, adjustment for potential confounders was carried out using the change-in-estimate method^21^. Covariables with *p*-value < 0.2 were introduced consecutively into the model, and those that modified the value of the measure of effect by at least 10% were retained in the final model. We consider as potential confounders the following variables and all of them were retained in the final model: gender, parental smoking habits, maternal education level, cat and dog exposure, and obesity.

The children with incomplete data were excluded from the study. The statistical analysis was performed using IBM SPSS statistics 24 software.

## Results

In the 6–7 year-old group, 284 centers were randomly selected, out of which 253 agreed to take part in the study. In the 13–14 year-old group, 143 schools were randomly selected, out of which 123 collaborated.

A total of 10,690 valid questionnaires were finally obtained in the 6–7 years age group, and 10,730 in the 13–14 years group, with a response rate of 72.4% and 84.4%, respectively.

The prevalence of rhinitis in the 6–7 years age group was: 29.4% for rhinitis ever, 24% for recent rhinitis, 11.5% for rhinoconjunctivitis, and 0.1% for severe rhinoconjunctivitis. In the group aged 13–14 years, the prevalence was 46.2%, 34.5%, 16.2%, and 0.2%, respectively. The prevalence of wheezing ever in the lower age group was 39.0%, that of current asthma was 13.5%, severe asthma 4.9%, and EIA 6.4%. In the 13–14 years old group, these prevalence’s were 23.0%, 13.2%, 5.8%, and 20.0%, respectively (Table 1). The general characteristics of the children, parental smoking, maternal education level, cat and dog exposure are shown in Table 2.

**Table 1:** Prevalence of rhinitis and asthma symptoms.

	6–7 years old		13–14 years old	
	*n*	%	*n*	%
**Rhinitis ever**				
No	7546	70.6	5775	53.8
Yes	3144	29.4	4955	46.2
Recent* rhinitis				
No	8128	76.0	7032	65.5
Yes	2562	24.0	3698	34.5
Recent* rhinoconjunctivitis				
No	9460	88.5	8993	83.8
Yes	1230	11.5	1737	16.2
**Severe rhinoconjunctivitis**				
No	10,680	99.9	10,713	99.8
Yes	10	0.1	17	0.2
**Wheezing ever**				
No	6519	61.0	8262	77.0
Yes	4171	39.0	2468	23.0
**Current asthma**				
No	9249	86.5	9319	86.8
Yes	1441	13.5	1411	13.2
**Exercise induced asthma**				
No	10,010	93.6	8585	80.0
Yes	680	6.4	2145	20.0
**Severe asthma**				
No	10,170	95.1	10,106	94.2
Yes	520	4.9	624	5.8

*n*: number of cases. *Recent refers to the previous 12 months.

**Table 2:** Main characteristics of the children and prevalence of risk factors.

	6–7 years old		13–14 years old	
	*n*	%	*n*	%
**Gender**				
Female	5270	49.8	5269	49.7
Male	5321	50.2	5323	50.3
**Parental smoking**				
Neither parent	5020	48.7	5057	48.4
Father only	1941	18.8	1893	18.1
Mother only	1354	13.1	1487	14.2
Both parents	1999	19.4	2016	19.3
**Obesity**				
Normal weight	5261	67	7421	82.3
Overweight	1834	23.4	1396	15.5
Obesity	752	9.6	197	2.2
**Cat at home in the past 12 months**				
Yes	773	7.4	1722	16.2
No	9699	92.6	8903	83.8
**Cat at home in the first year of life**				
Yes	584	5.5	879	11.6
No	10,026	94.5	6730	88.4
**Dog at home in the past 12 months**				
Yes	1313	12.6	3050	28.7
No	9111	87.4	7563	71.3
**Dog at home in the first year of life**				
Yes	1123	10.6	1559	20.5
No	9457	89.4	6060	79.5
**Maternal education level**				
No schooling/elementary	2981	28.4	2261	22.1
High school	4012	38.2	4418	43.2
University	3519	33.5	3553	34.7

*n*: number of cases.

Univariate analysis shows that the prevalence of asthma symptoms was significantly elevated in the patients with any of the forms of rhinitis analysed (Table 3). Considering the low prevalence of severe rhinoconjunctivitis, the relationship with asthma symptoms were not analysed in these cases. The highest prevalence of asthma symptoms was observed in the patients with recent rhinoconjunctivitis, while there was wheezing ever in 74.4% of children in the 6–7 years group, and in 45.1% of the 13–14 years old group (Table 3).

**Table 3:** Prevalence of asthma symptoms according to rhinitis.

	Wheezing ever		*p*	Current asthma		*p*	Exercise-induced asthma		*p*	Severe asthma		*p*
	No	Yes		No	Yes		No	Yes		No	Yes	
**6–7 years old**												
Recent rhinoconjunctivitis												
No	6204 (65.6)	3256 (34,4)	0.000	8585 (90.8)	875 (9.2)	0.000	9123 (96.4)	337 (3.6)	0.000	9203 (97.3)	257 (2.7)	0.000
Yes	315 (25.6)	915 (74.4)		664 (54.0)	566 (46.0)		887 (72.1)	343 (27.9)		967 (78.6)	263 (21.4)	
Rhinitis once												
No	5323 (70.5)	2223 (29.5)	0.000	7037 (93.3)	509 (6.7)	0.000	7385 (97.9)	161 (2.1)	0.000	7428 (98.4)	118 (1.6)	0.000
Yes	1196 (38.0)	1948 (62.0)		2212 (70.4)	932 (29.6)		2625 (83.5)	519 (16.5)		2742 (87.2)	402 (12.8)	
Recent rhinitis												
No	5595 (68.8)	2533 (31.2)	0.000	7550 (92.9)	578 (7.1)	0.000	7933 (97.6)	195 (2.4)	0.000	7987 (98.3)	141 (1.7)	0.000
Yes	924 (36.1)	1638 (63.9)		1699 (66.3)	863 (33.7)		2077 (81.1)	485 (18.9)		2183 (85.2)	379 (14.8)	
**13–14 years old**												
Recent rhinoconjunctivitis												
No	7308 (81.3)	1685 (18.7)	0.000	8157 (90.7)	836 (9.3)	0.000	7572 (84.2)	1421 (15.8)	0.000	8690 (96.6)	303 (3.4)	0.000
Yes	954 (54.9)	783 (45.1)		1162 (66.9)	575 (33.1)		1013 (58.3)	724 (41.7)		1416 (81.5)	321 (18.5)	
Rhinitis once												
No	4880 (84.5)	895 (15.5)	0.000	5335 (92.4)	440 (7.6)	0.000	5045 (87.4)	730 (12.6)	0.000	5613 (97.2)	162 (2.8)	0.000
Yes	3382 (68.3)	1573 (31.7)		3984 (80.4)	971 (19.6)		3540 (71.4)	1415 (28.6)		4493 (90.7)	462 (9.3)	
Recent rhinitis												
No	5854 (83.2)	1178 (16.8)	0.000	6475 (92.1)	557 (7.9)	0.000	6057 (86.1)	975 (13.9)	0.000	6832 (97.2)	200 (2.8)	0.000
Yes	2408 (65.1)	1290 (34.9)		2844 (76.9)	854 (23.1)		2528 (68.4)	1170 (31.6)		3274 (88.5)	424 (11.5)	

Univariate analysis.

Data shown as number of cases (*n*) and percentage (%); *Recent refers to the previous 12 months.

In the multivariate analysis it was confirmed that rhinitis was associated with a significant increase in the prevalence of asthma, achieving an OR of 11.375 (9.233–14.014) for EIA in 6–7 years old children with recent rhinoconjunctivitis and 9.807 (7.612–12.635) for children of this same age group with recent rhinitis. In general, the prevalence OR’s are higher in the 6–7 years old group than in the 13–14 years old group, especially for EIA and severe asthma (Table 4).

**Table 4:** Prevalence of asthma symptoms according to rhinitis. Multivariate analysis.

	Wheezing ever	Current asthma	Exercise-induced asthma	Severe asthma
	OR (CI 95%)	OR (CI 95%)	OR (CI 95%)	OR (CI 95%)
**6–7 years old**				
Recent rhinoconjunctivitis				
No	1	1	1	1
Yes	5.584 (4.726–6.599)	8.797 (7.468–10.363)	11.375 (9.233–14.014)	9.788 (7.749–12.365)
Rhinitis once				
No	1	1	1	1
Yes	3.946 (3.544–4.392)	5.874 (5.079–6.793)	9.291 (7.402–11.661)	9.358 (7.181–12.195)
Recent rhinitis				
No	1	1	1	1
Yes	3.913 (3.489–4.388)	6.710 (5.798–7.765)	9.770 (7.856–12.152)	9.807 (7.612–12.635)
**13–14 years old**				
Recent rhinoconjunctivitis				
No	1	1	1	1
Yes	3.492 (3.009–4.054)	4.428 (3.730–5.257)	3.564 (3.059–4.152)	6.420 (5.057–8.152)
Rhinitis once				
No	1	1	1	1
Yes	2.539 (2.233–2.887)	2.917 (2.466–3.450)	2.617 (2.285–2.998)	3.317 (2.556–4.305)
Recent rhinitis				
No	1	1	1	1
Yes	2.638 (2.324–2.995)	3.299 (2.810–3.874)	2.655 (2.325–3.031)	4.310 (3.364–5.522)

OR: Odds Ratio; CI: Confidence Interval; *Recent refers to the previous 12 months.

Adjusted by: gender, maternal education, body mass index, parental smoking, cat and dog at home.

## Discussion

The prevalence of rhinitis is elevated in the community studied, exceeding that present in most Western Europe countries that have available data obtained with the same methodology^18^. In the studied population, rhinitis is clearly more prevalent in the adolescents, unlike what happens in asthma in this same population, where a higher prevalence is observed in the 6–7 years old group, except for EIA^20^. In our area, asthma is a disease with an elevated prevalence in children and adolescents with rhinitis, as more than half of the 6–7 years old children and more than one-third of the 13–14 years old group have asthma symptoms. This agrees with the results of a recent meta-analysis that showed a strong association between both diseases^22^. The impact seems particularly relevant in severe asthma, as the presence of rhinitis appears to increase the prevalence of severe asthma by between 3 and 6 times in the older aged group, and more than 9 times in the younger age group. This relationship between rhinitis and more severe forms of asthma has been noted in children as well as adults by other authors^8,9,23–25^; although other authors did not observe any relationship between the rhinitis and the severity of the asthma^11,26,27^.

This association between rhinitis and asthma may be due to several factors. On the one hand, the rhinitis, by itself, may aggravate the asthma since the asthmatics with rhinitis have a higher FeNO, a lower FEV1, and greater bronchial hyper-reactivity^23,25,28^. On the other hand, there could be factors, both genetic and environmental, that may contribute to aggravating both the asthma and the rhinitis. It is known that allergic sensitisation, **f**ilaggrin loss-of-function mutations and other genetic alterations, treatments, maternal nutrition during pregnancy, and environmental exposure^12,29–36^.

Another aspect to consider is the concept of ‘united airway diseases (UAD), as such that the upper airway and the lower airway form a single organ, and the symptomatology would be a reflection of the same underlying process with some clinical, epidemiological, and pathophysiological differences^37^. Some mechanisms have been established that appear to support the pathophysiological interactions between the upper and lower airway, by conditioning the air that we breathe, common inflammatory processes and neural reflexes. The exposure to allergens produces a series of IgE reactions mediated by the release of mastocyte and basophil mediators that would produce rhinorrhoea and sneezing in the upper airway, as well as a cough, oedema, mucosa secretion in the lower airway. At the same time, in the post-mortems of humans that have died due to asthma, it has been demonstrated that there is eosinophilic inflammation in the whole respiratory tract from the nasal mucosa to the lung tissue. Furthermore, the nasal-bronchial reflex is well documented in animal models, which produces an increase in the resistance in the lower airway after nasal provocation with cold air or allergens^12^.

In conclusion, there is an elevated prevalence of rhinitis in children and adolescents in our community, and it seems to be associated with higher prevalence and severity of the asthma.
